# Controlling Maize Weevil (*Sitophilus zeamais*) Life Stages at a Moderate Temperature Using Microwave Energy

**DOI:** 10.3390/insects17010017

**Published:** 2025-12-23

**Authors:** Amro B. Hassan, Dieter von Hoersten, Shaojin Wang

**Affiliations:** 1 Department of Food Science and Nutrition, Faculty of Food and Agricultural Sciences, King Saud University, P.O. Box 2460, Riyadh 11451, Saudi Arabia; 2 Institute for Application Techniques in Plant Protection, Julius Kühn Institute, Federal Research Centre for Cultivated Plants, 38104 Braunschweig, Germany; 3 College of Mechanical and Electronic Engineering, Northwest A&F University, Yangling 712100, China; 4 Department of Biological Systems Engineering, Washington State University, Pullman, WA 99164-6120, USA

**Keywords:** microwave, maize, maize weevil, mortality, germination, validation

## Abstract

The objective of this study is to evaluate the effect of microwave energy in controlling life stages (egg, larvae, pupa, and adults) of maize weevil (*Sitophilus zeamais*) and validate the optimum condition for eliminating the pest in maize grain. Maize samples (300 g) each at 10, 14, and 18% w.b. initial moisture contents (IMCs) were infested with various life stages of *S. zeamais* and exposed to microwave energy (2.45 GHz) at 300 W to raise the grain’s temperature to 50 °C, holding for different times of 0, 1, 3, and 5 min. Among the life stages of *S. zeamais*, the egg stage was the most susceptible to microwave energy, whereas the adult stage was the least susceptible. Complete eradication of all life stages of *S. zeamais* can be achieved by using microwave energy at a temperature of 50 °C for 5 min at all initial moisture content levels. Germination rate was significantly (*p* < 0.05) lower in the microwave-treated grains compared to the untreated ones. The partial least squares (PLS) validation model indicated that microwave heating of grain with 14% moisture content at 50 °C for 1 min was the most effective method for eliminating *S. zeamais* life stages and maintaining the germination rate of the grain, which could be further explored for industrial applications in the maize processing.

## Simple Summary

As an eco-friendly, non-chemical method, microwave energy presents a promising alternative for the phytosanitary treatment of grain. Microwave heating is considered an efficient and rapid dielectric heating method for eliminating pests in various major grain-based stored products. Several factors affect the efficiency of microwave energy. The moisture content of the grains plays an important role in the effective application of microwave as a controlling method. Therefore, in this work, microwaves were applied at a moderate temperature of 50 °C to control the life stages of maize weevils in maize with different initial moisture contents of 10, 14, and 18%, respectively. The results showed that all maize weevil life stages were eliminated at 50 °C for 3 to 5 min; however, the most effective condition for controlling the pests and maintaining grain quality, in terms of germination rate, was found to be maize grain (14% MC) treated at 50 °C for 1 min. This condition can be further explored for industrial applications in maize processing.

## 1. Introduction

A major problem in the production, processing, and storage of maize is insect infestation. It is assessed as accountable for up to 36% of postharvest losses in maize and other cereal crops in tropical and subtropical areas, Suleiman et al. [1]. The maize weevil, *Sitophilus zeamais* (Coleoptera: Curculionidae), is one of the most economically significant pests of stored cereal grains, particularly maize. In stored maize, heavy infestation of *Sitophilus zeamais* may cause different types of damage, which have a negative impact not only on the quantity but also on quality of grains [2]. Generally, chemical fumigants are used to disinfest grains [3]; however, widespread use of some pesticides results in a negative impact on human health and the environment [4]. Therefore, the grain industry has been forced to explore non-chemical alternatives. One possible alternative is the use of dielectric heating and microwave (MW) energy to control insect pests in agricultural commodities. Microwaved (300 MHz to 300 GHz) are non-ionizing electromagnetic waves that generate heat in dielectric materials, such as grains, through dipole rotation and/or ionic polarization [5].

Several studies have investigated the application of microwave energy on controlling stored-grain insects, such as *Tribolium castaneum* in wheat [6], barley [7], and rye [8], *Oryzaephilus surinamensis* (L.) in soft winter wheat [9], *Sitophilus oryzae* L. in rice [10], *Sitophilus granarious* in wheat [6], and *Collosobruchus chinesis* in pulses [11]. They concluded that a higher rate of microwave heating with a shorter exposure time would be effective in controlling insects completely.

Controlling insects of stored products by using microwave energy depends mainly on the differences in the dielectric properties of the insect’s body and grains [12]. However, the dielectric properties of materials depend on many factors, including the frequency, moisture content, and many other factors, but mainly on the moisture content. Furthermore, it was noted that increasing the moisture content of the material raised its heating rate during microwave treatment. Therefore, it is possible to heat the insects to the lethal temperature at a faster rate than infested products, since they contain relatively higher water content [13]. Moreover, the mortality of the insects is also likely influenced by the moisture content of the grains. Therefore, it is important to determine the appropriate microwave parameters for developing the effective disinfestation treatment protocol of maize grains.

Generally, the strategies for the thermal treatments for grains disinfestation require an optimum combination of treatment time and temperature that will cause a high mortality rate of a target insect and also maintain commodity quality. Hence, the objectives of the present investigation were to study the effectiveness of microwave energy under moderate temperate (50 °C) in controlling life stages of maize weevil, *Sitophilus zeamais*, in maize grains at different initial moisture contents (IMCs) of 10, 14, and 18% w.b. and the moderate temperature of 50 °C for different holding times of 0, 1, 3, and 5 min, as well as to evaluate the germination capacity of microwave-treated maize.

## 2. Materials and Methods

### 2.1. Grain and Insects Culture Preparation

Maize grains (*Zea mays* L.) cultivar “Amadeo” used in this study were obtained from KWS SAAT AG, Einbeck, Germany. The moisture content of the grains was modified to three levels, 10, 14, and 18% w.b., by adding a calculated amount of water to the grains and then mixed with a rotator shaker at room temperature for 5 h. After that the grains were kept in a refrigerator for 72 h at 4 °C. During this period the grains were mixed 4 to 5 times per day in order to obtain uniform moisture content. The final moisture content was measured according to the AOAC [14]. Maize weevil adults, *Sitophilus zeamais* (Coleoptera: Curculionidae) culture was obtained from Julius Kühn Institute, Berlin, Germany. The insects were reared in a laboratory climate chamber in 12% w.b. moisture content on whole maize at 28 °C and 70% relative humidity (r.h) [2].

### 2.2. Identification of S. zeamais Immature Life Stages

To obtain immature life stages, 5 g maize grains with 12% w.b. were infested with 100 unsexed adults of *S. zeamais*, and the infested grains were incubated in an environmental chamber for 72 h at 28 °C and 70% r.h under controlled light/darkness period (12 h light/12 h dark). After that, the adults were removed, and the kernels were immersed in acid fuchsin to stain the egg plugs in order to determine the infested kernels according to Frankenfield [15]. Maize grains infested for 3 d contained the egg stage, and the rest of the infested samples were incubated for 20 and 27 d to obtain larva and pupa stages, respectively.

### 2.3. Microwave System

A laboratory microwave system operating at a frequency of 2.450 GHz and output power of 300 W was used in this study. The microwave applicator consists of a magnetron (Muegge, Electronic MW-GIRYJ1540-1k2-08500, Reichelsheim, Germany), a wave guide, and a cavity. The microwave cavity with the dimensions of 34.5 × 22.5 × 34 cm^3^ had a special rotating Teflon sample plate which was suspended in an electronic balance. The sample plate could be adjusted in height and was coupled with a rotary device under rotation speeds of 30 rpm, and also with a sensitive balance to measure the sample weight during the treatment. An infrared pyrometer (Heimann KT 19.82, Heimann GmbH, Dresden, Germany) was installed on the top of the cavity to measure the surface temperature of the grains during microwave treatment.

### 2.4. Grain Infestation and Microwave Treatment

Maize grains, 300 g, were infested with 20 individuals of *S. zeamais* for each life stage bound in plastic wrap during the treatments. The infested grains were placed on the rotating Teflon plate in a single kernel layer, and then the rotation speed of the sample plate was set at 30 rpm. In this study, two factors that influence microwave heating were evaluated. These factors included initial moisture contents of grains (10, 14, and 18% w.b.), and holding times (0, 1, 3, and 5 min after reaching the target temperature). After preparation, the infested grains were then exposed to the microwave energy at 300 W to raise the grains’ temperature to 50 °C for different holding times of 0, 1, 3, and 5 min.

### 2.5. Assessment of the Insect’s Mortality

#### 2.5.1. Mortality of Adults

The mortality of the adults was determined by counting the number of living and dead adults. Adults were counted in 24 h after exposure to microwave energy. The mortality percentage was calculated as described by Zhao et al. [10] in counting the number of dead insects, and expressing it as

Ma = Nd/Ns × 100

where Ma is the mortality percent of adults (%), Nd is the number of dead adult insects for each trial, and Ns is the sum of live and dead adult insects for each trial.

#### 2.5.2. Mortality of Immature Life Stages

Since *S. zeamais* could complete development within kernels, the effectiveness of microwave can be ascertained by examining the number of adults that emerged in treated maize relative to emergence in untreated maize. The mortality of the eggs, larvae, and pupae was determined based on the number of adults which emerged in sample for each trial after 35, 15, and 10 d, respectively. The mortality percentage of all immature life stages was calculated separately using the following equation described by Zhao et al. [10]:

Mi = (1 − Nt /Nc) × 100

where Mi is the mortality percentage of the immature life stage (%), Nt is the number of adults that emerged for each trial, and Nc is the number of adults that appeared in the control sample.

### 2.6. Determination of Germination

The germination rate of maize grains subjected to microwave heating was determined following the between-paper (BP) procedure from the International Seed Testing Association [16]. The seeds (100) were kept at 25 °C for 7 d. On the seventh day, the germinated seeds were counted, and the germination percentage was calculated.

### 2.7. Statistical Analysis and Multivariable Analysis

All data were described by the mean and standard deviation over triplicate. Data were analyzed using two-way analysis of variance (ANOVA). Significant differences were calculated (*p* < 0.05) using least significant difference (LSD). Multivariable analysis, principal component analysis (PCA), and partial least squares regression (PLS) were implemented in XLSTAT software [17,18].

## 3. Results

### 3.1. Mortality of Sitophilus zeamais

**Figure 1:**
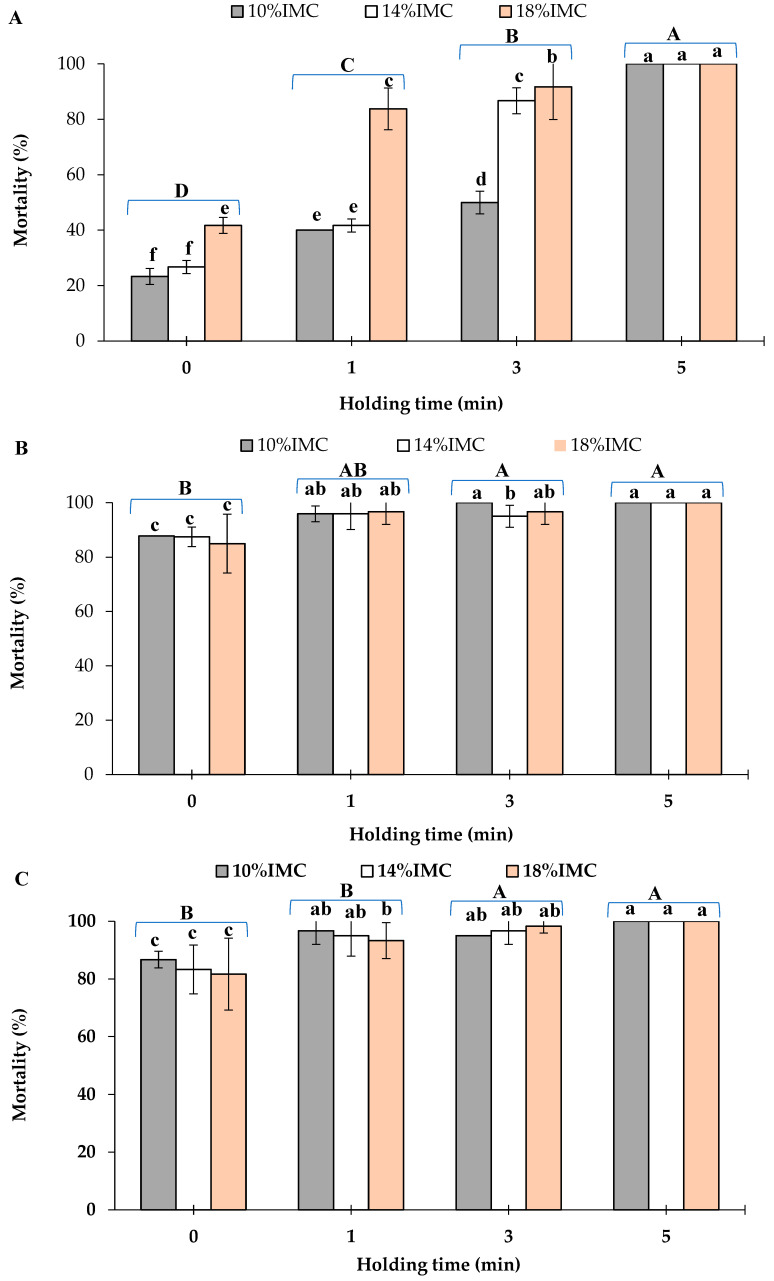

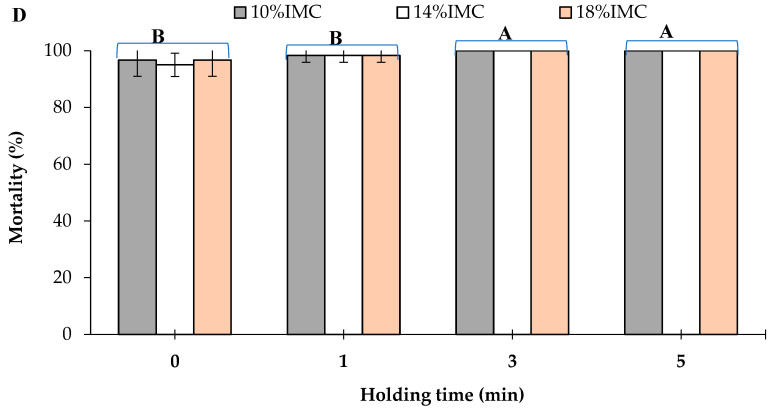
Mortality of *Sitophilus zeamais* life stages of (**A**) adults, (**B**) pupae, (**C**) larvae, and (**D**) eggs after microwave heating at 50 °C for different holding times in maize grains at different initial moisture contents (IMCs). Data represent the mean ± SD (n = 3). Values without the same letter are not significantly different. Small caps represent the difference between IMC, and the capital caps represent the difference among the application time as assessed by LSD.

**Figure 2:**
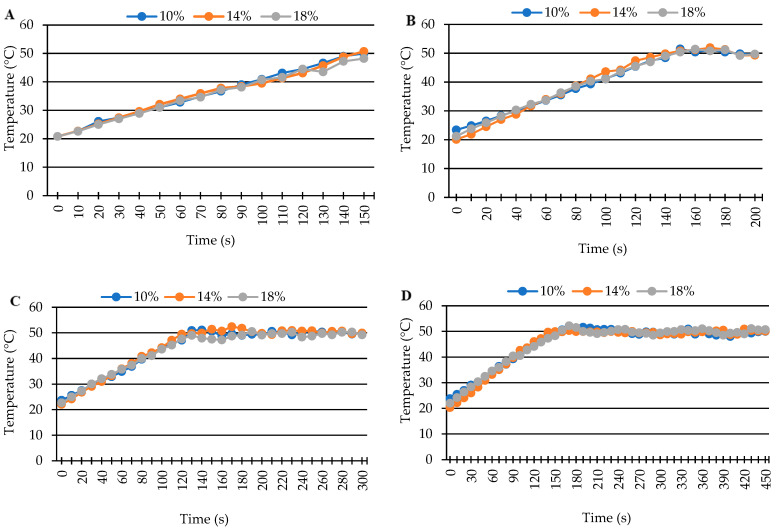
Temperature–time history profile during microwave heating of maize at 50 °C for 0 min (**A**), 1 min (**B**), 3 min (**C**), and 5 min (**D**) holding times.

Figure 1A–D shows the mortality rates of *S. zeamais* life stages in maize grains at various IMCs at 50 °C for different holding times: 0, 1, 3, and 5 min. In Figure 2A, it was clearly observed that the mortality of *S. zeamais* adults was significantly (*p* < 0.001) influenced by both the holding times and the IMCs. The mortality rate significantly (*p* < 0.001) increased with the increase in holding time and IMCs of the grains. Furthermore, one hundred per cent of adults’ mortality was obtained when the temperature (50 °C) was held for 5 min for all levels of IMCs. As shown in Figure 1B–D, for all levels of the IMCs, the mortality rate of *S. zeamais* immature stages, pupa, larva, and egg was higher than 80% when the infested grains were treated with the microwave at 50 °C. Similarly to the adults, prolongation of the holding time significantly (*p* < 0.05; *p* < 0.001; *p* < 0.05) raised the mortality of the pupa, larva, and egg stages, respectively (Table 1). Furthermore, holding time for 3 min completely eradicated the eggs, while one hundred per cent of larvae and pupae mortality was obtained when the holding time was 5 min at 50 °C for all IMCs levels. However, at 50 °C, the comparisons of means indicated that both holding times and IMCs had no significant influence on the mortality of pupa and egg stages (Table 1).

### 3.2. Effect of Microwave Heat Treatments on the Germination Rate of Maize Grains

**Figure 3:**
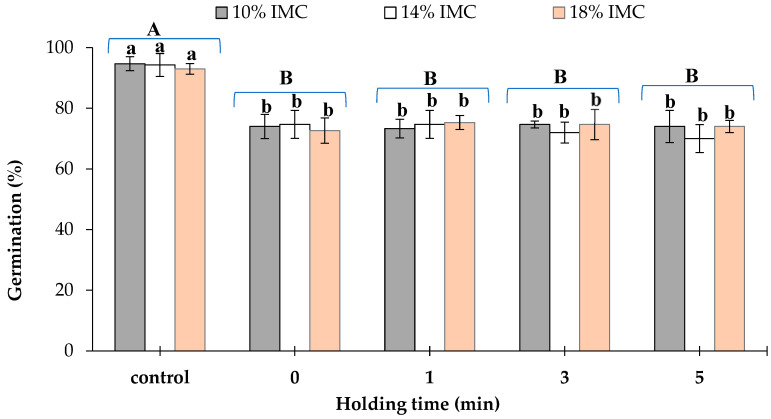
Germination rates of maize grains after microwave heating at 50 °C for different holding times in maize grains at different initial moisture contents (IMCs). Data represent the mean ± SD (n = 3). Values followed by the same letter are not significantly different. Small caps represent the difference between IMC, and the capital caps represent the difference among the application time as assessed by LSD.

Before microwave treatments, the initial germination rates of the maize grains were found to be 94.7, 94.3, and 93% for the grains at 10, 14, and 18% w.b. IMCs, respectively. As already mentioned, the germination rate of maize grains was significantly influenced by the temperature. Treatment of the grains with microwave at temperature of 50 °C significantly (*p* < 0.001) reduced their germination rate to 74.0, 74.7, and 72.67% for grains at 10, 14, and 18% w.b. IMCs, respectively (Figure 3). However, prolongation of the holding time up to 5 min at the target temperature 50 °C had insignificant (*p* < 0.05) influence on the germination rate of the grains, which remained in the same range, between 70.0 and 75.0%. Furthermore, the comparison of means showed that the germination rate was similar among 10, 14, and 18% w.b. IMCs, respectively (Figure 3).

### 3.3. Multivariate Statistics

**Figure 4:**
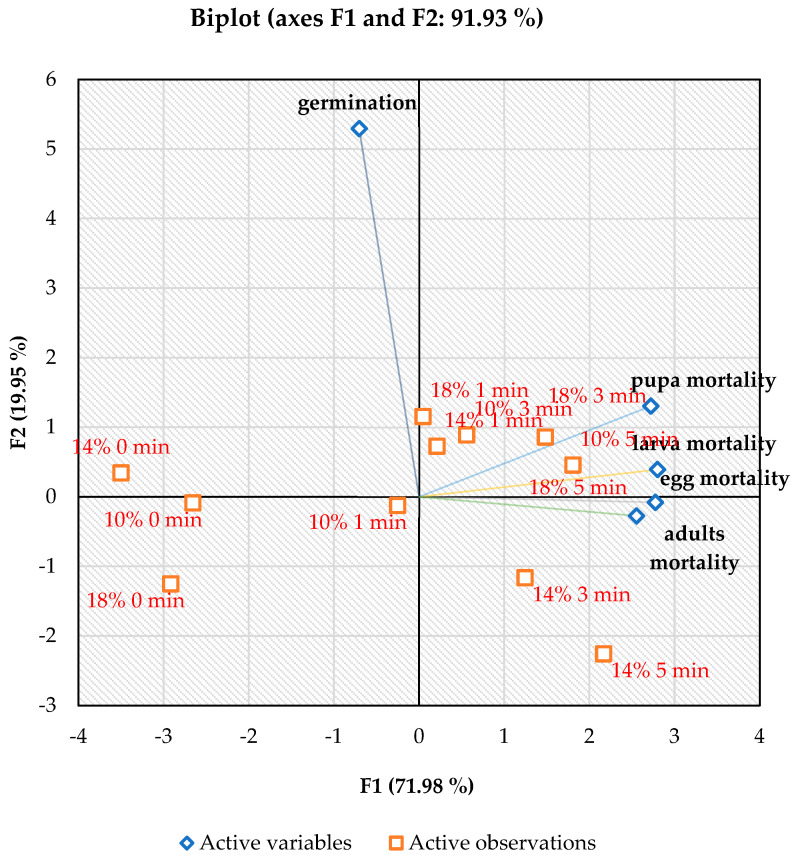
Principal component analysis for the parameters determined during microwave heat treatments.

**Figure 5:**
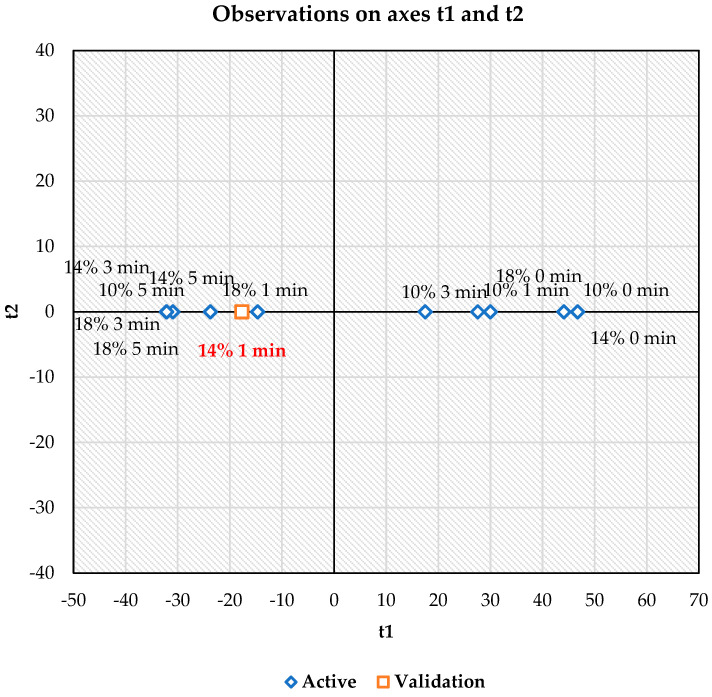
Partial Least Squares regression analysis for the parameters determined during microwave heat treatments.

The multivariate statistics demonstrated the relationship between microwave treatment and the mortality rate of maize weevil life stages and germination rate of the maize grains. The multivariable analyses were shown using PCA (Figure 4) and PLS (Figure 5). Regarding PCA, the axes involving PC1 and PC2 explain 71.98% and 19.95% of the variance, respectively, with an overall variability of 91.93% for the plotted components. The biplot revealed a positive relationship between the microwave treatment and holding time (1–5 min at 50 °C) and the mortality rate of all maize weevil life stages, as shown in the third and fourth quadrants of the plot. Conversely, the impact of the microwave energy on the mortality rate was dispersed across the first and second quadrants. Additionally, a positive relationship was observed among microwave-treated samples with 10, 14, and 18 w.b. MC at 50 °C for 1, 3, and 5 min and the variables of mortality rate, as described by Yan et al. [19], who stated that variables occurring at <90° angles might be positively correlated.

The PLS validation model is presented in Figure 5. PLS demonstrated the interaction effect of microwave treatments (x variables) on the mortality rate of maize weevil life stages. As depicted, the PLS confirmed that application of microwave energy to maize grain with 14% MC at 50 °C for 1 min represented the most strategic treatment for improving the mortality rate of maize weevil and maintaining the germination level of the maize grain, which could be considered for further applications in eliminating pests in maize.

## 4. Discussion

In this study, the data show that using microwave heating at a moderate temperature (50 °C) was possible to completely eradicate *S. zeamais* stages. Additionally, the lethal combination of temperature and holding time of *S. zeamais* in maize grains at 10, 14, and 18% IMCs were estimated as 50 °C and 3 min for its immature life stages, whereas they were found to be approximately 50 °C after 5 min for the adults at the IMCs of 10% and 14% IMC. According to Rees [2], females of *S. zeamais* lay a single egg inside the kernel, and thus all developments of the immature stages are completed inside the kernel. Since microwaves penetrate and generate heat from the inner side of the dielectric materials (such as maize kernels), the temperature inside the kernel may be higher than the surface temperature. Therefore, the mortality rates of the immature life stages were higher than those of the adults. Similar findings were observed in the application of radio frequency (RF) energy in controlling maize weevil. Ramírez-Rojas et al. [20] reported that RF heating at 50 °C for 3 min results in complete eliminating of maize weevil in white maize.

Since the dielectric properties of grains vary with their moisture content [21,22], by using microwave heating in the present study, the mortality results showed that the IMCs of the grains significantly (*p* < 0.05) influence the mortality rate of *S. zeamais*, especially at the moderate temperature. During microwave treatments, comparison of means showed that *S. zeamais* eggs were not significantly (*p* < 0.05) different among the grains at 10, 14, and 18% w.b. IMCs, whereas, for larvae and pupae, the mortality rate was significantly (*p* < 0.05) higher in the grains at 10% w.b. IMC than at 14 and 18% w.b. These findings probably would be due to the penetration depth of the microwaves. It was noted that the penetration depth of microwaves decreased with the increasing moisture content [23], frequency, and temperature [24]. Decrease in the penetration depth in the moist grains would explain the higher mortality rates of *S. zeamais* immature stages in the grains at 10% w.b. IMC than in grains at 14 and 18% w.b. IMC, since these stages developed within the grain [2].

In contrast, for the adult, the mortality had a reversed pattern compared to the other stages. The mortality rate significantly (*p* < 0.05) increased as the initial moisture content of maize increased. A higher mortality rate of the adults was achieved when they were treated with maize grains at 18% w.b. IMC. These findings can be explained by the character of the dielectric properties of the agricultural material, especially the dielectric loss factors, which increased with increasing moisture content. Moreover, the heating rate of grains also increased as the moisture content increased [23].

The purpose of testing various ages of insects to microwave heating was to identify the most heat-tolerant stage, because controlling the most heat-tolerant stage may control all other stages. In our study, the heat sensitivity was varied among the different developmental stages of *S. zeamais*. The egg was the most susceptible stage, whereas the adults were the most resistant ones to microwave heating. The variation in susceptibility among *S. zeamais* stages may be related to the adverse effects of microwave energy on the insect’s physiological processes. Moreover, the location of the insect within the kernel also may influence its susceptibility to heat [25]. Results obtained in this study are similar to those obtained by Vadivambal et al. [6,7] who reported that among different life stages of red flour beetle, *Tribolium castaneum*, the egg stage was the most susceptible to microwave energy, while the adults were the least susceptible ones. Furthermore, Zhao et al. [10] reported that eggs of rice weevil are more susceptible to microwave energy than the adults.

The obtained results indicated that the temperature significantly (*p* < 0.05) influenced the germination rates of maize grain. These results agreed with those reported by Hassan et al. [26], who concluded that microwave heat treatment of corn at 10, 14, and 18% initial moisture content at 50, 55, and 60 °C significantly influenced the germination rates. These findings agree with the observations of Vadivambal et al. [27] who studied the germination of corn seeds at 14, 16, and 18% moisture content after microwave heating. Reductions in the germination rate of maize grain after microwave treatments may be due to the generation of high temperatures within the grain’s germ. It has been stated that the germ of maize grain contains about 83% oil [28]. Hence, due to its higher dielectric properties than other parts, it may absorb more microwave energy, leading to elevated temperatures and denaturation of the germ’s protein responsible for germination.

## 5. Conclusions

The present study demonstrates the feasibility of employing microwave energy as an alternative method to chemical fumigants in controlling maize weevil, *Sitophilus zeamais*, in maize grains. Application of microwave energy at moderate temperature of 50 °C for 3–5 min caused a complete eliminating of all developmental stages of *S. zeamais*. However, the germination rate of the grain was significantly reduced by exposure to microwave energy at 50 °C. From the PCA it was confirmed that microwave energy had a significant effect on the mortality rate of *S. zeamais* life stages. Moreover, the validation model, PLS, verified the strategic combination of temperature (50 °C) and holding time (1 min) in application of microwave energy in controlling maize weevil in maize grain (14% IMC), and maintaining their germination rate and could be advantageous for future food processing. However, further studies are needed on the application of different microwave powers to improve their efficiency to reach a reasonable usage in controlling stored-grain insects.

## Figures and Tables

**Table 1:** Two-way ANOVA analysis of the effect of time and IMC on the mortality of the life stages of maize weevil and germination of maize grains.

	Factors	Mortality of Life Stages				Germination
		Adult	Pupa	Larva	Egg	
	**Time**	249.23 ***	3.129 *	12.481 ***	4.532 *	35.788 ***
**F-values**	**IMC**	64.865 ***	ns	12.481 ***	ns	ns
	Time*IMC	17.355 ***	ns	5.062 **	ns	ns
	**Time SE±**	1.875	1.860	1.479	1.076	1.609
**SE values**	**IMC SE±**	3.248	1.611	1.281	0.932	1.246
	Time*IMC SE±	3.248	3.222	2.562	1.863	2.786

The data were evaluated using a two-way ANOVA, and the factors were time and initial moisture content of maize grains. Asterisks * significant at *p* < 0.05; ** significant at *p* < 0.01; *** significant at *p* ≤ 0.001 level. ns, insignificant.

## Data Availability

The original contributions presented in this study are included in the article. Further inquiries can be directed to the corresponding author.
